# Small modifier, big decision: switching to SUMO mode adds weight to cancer stemness in mammary tumors

**DOI:** 10.1002/1878-0261.70082

**Published:** 2025-06-23

**Authors:** Veronika Yevdokimova, Yannick D. Benoit

**Affiliations:** ^1^ Department of Cellular and Molecular Medicine Faculty of Medicine, University of Ottawa Canada; ^2^ School of Pharmaceutical Sciences Faculty of Medicine, University of Ottawa Canada

**Keywords:** breast cancer, cancer stem cells, chromatin regulation, drug discovery, SUMOylation

## Abstract

Protein SUMOylation is crucial for maintaining the hallmarks of cancer stem cells, including self‐renewal and active pluripotency gene networks. While inhibiting key steps of the SUMOylation cascade has been shown to suppress tumorigenesis, the specific mechanisms of SUMO dependency in cancer have not been comprehensively characterized. Li *et al*. applied genetically engineered models of mammary gland tumorigenesis to demonstrate that SUMOylation of the transcription factor Etv1 is essential for maintaining cancer stem cell functions. Moreover, SUMO conjugation of Etv1 acts as a switch between stem and nonstem cancer cell states. Here, we discuss the implications of these findings regarding the role of SUMOylation‐dependent mechanisms in the hierarchical organization of malignant cells and intratumor heterogeneity and highlight potential therapeutic approaches harnessing the SUMOylation cascade.

## Graphical abstract

Inhibition of protein SUMOylation has been shown to block tumorigenesis; however, the specific mechanisms by which SUMOylation controls the tumor‐initiating capacities remain elusive. Li *et al.* describe the role of Etv1 SUMOylation in cancer stem cells using mouse models of mammary gland tumorigenesis. Here, we discuss the implications of these findings and highlight potential anticancer therapeutic approaches targeting this cascade.

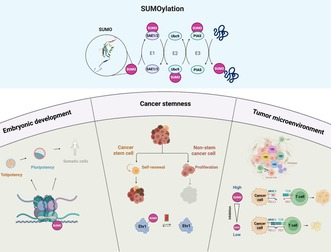

**Abbreviations**
CSCs: cancer stem cells
ESCs: embryonic stem cells
Etv1: ETS variant transcription factor 1
H3K9: histone 3 lysine‐9
MHC‐I: major histocompatibility complex class 1
MMTV: mouse mammary tumor virus
PDAC: pancreatic ductal adenocarcinoma
PTM: post‐translational modification(s)
SENPs: sentrin‐specific proteases
SUMO: small ubiquitin‐like modifier
WNT: Wingless‐type MMTV integration site family

## 1 Protein SUMOylation is necessary for maintaining tumor‐initiating cell state

**Fig. 1:**
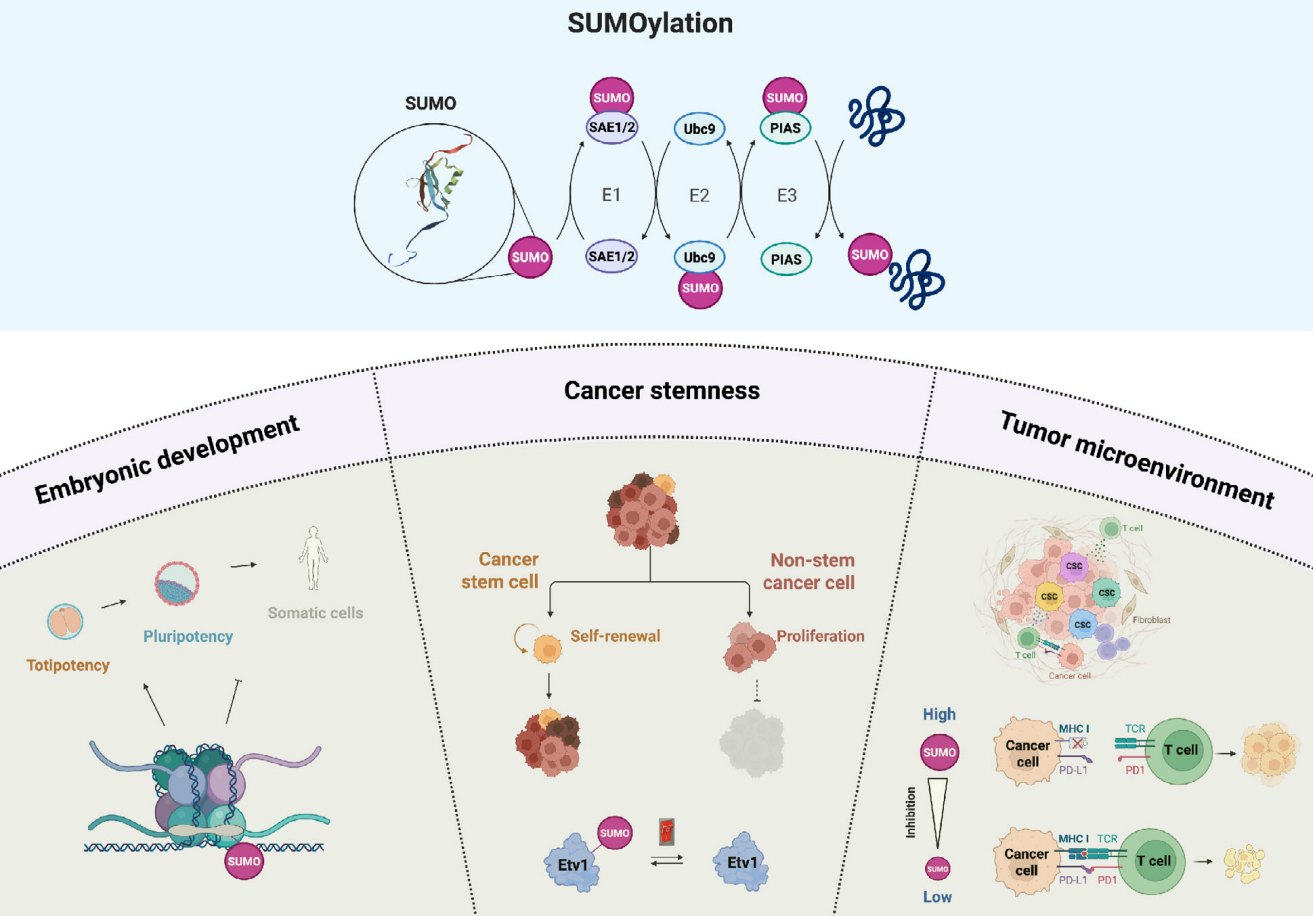
Protein SUMOylation is necessary for cell state transitions and tumorigenesis. In embryonic development, dynamic levels of SUMOylation control stem cell potency and differentiation. Cancer stem cells are maintained by high activity of the SUMOylation pathway, and SUMOylation of transcription factor Etv1 acts as a switch between stem and nonstem cancer cell states in mammary tumors. Moreover, SUMOylation‐dependent mechanisms in cancer cells also contribute to the establishment of a tumor‐permissive microenvironment.

SUMOylation is a three‐step enzymatic process involving an E1‐activating enzyme (SAE1/2), E2‐conjugating enzyme (Ubc9), and E3 ligases, influencing the activity, stability, or localization of target proteins (Fig. 1). Protein SUMOylation is a dynamic process that can be reversed by sentrin‐specific proteases (SENPs).

Multiple reports indicate that the SUMOylation machinery is activated in cancer, maintaining the tumor‐initiating capacity of cancer stem cells (CSCs). Pharmacological and genetic inhibition of SUMOylation has been shown to block tumorigenesis in models of breast, colorectal cancer, and acute myeloid leukemia [1, 2]. However, specific SUMO‐sensitive mechanisms responsible for tumor initiation and growth have remained elusive.

In an article published in *Developmental Cell*, Li *et al*. [1] shed light on a SUMOylation‐dependent mechanism essential to mammary gland tumorigenesis. The authors used a transgenic MMTV/Neu (Erbb2) mouse model of breast cancer to confirm that blockade of SUMOylation, using anacardic acid (E1‐SUMO intermediate inhibitor) or conditional knockout of Ube2i (encoding E2 enzyme Ubc9), extends tumor‐free survival with no apparent deficiencies in normal mammary gland development and stem cell activity. Single‐cell transcriptome profiling highlighted the expansion of a cluster of alveolar epithelium‐like cells in MMTV‐Neu mammary glands, which reverted to control proportions in MMTV‐Neu Ube2i knockout animals. This cluster was marked by the enrichment of ETS Variant Transcription Factor 1 (Etv1), a SUMOylation‐sensitive transcriptional driver of pro‐oncogenic pathways [3]. Functionally, Etv1 knockdown reduced proliferation and colony formation capacity in mouse and human breast cancer cells. Interestingly, ectopic restoration experiments using SUMO‐resistant (lysine‐to‐arginine) and constitutive SUMO‐conjugated (SUMO1/2 C‐terminal fusion, lysine‐to‐arginine) mutants of Etv1 differentially rescued the effects of Ube2i knockout. Following a series of *in vitro* and *in vivo* experiments, the authors proposed a paradigm in which ectopic SUMO‐conjugated Etv1 re‐establishes tumor‐initiating capacities in a SUMOylation‐deficient context but leaves tumor cells in a low‐cycling/quiescent state, akin to the CSC phenotype (Fig. 1). Conversely, transduction of Ube2i knockout cells with a SUMO‐resistant form of Etv1 exclusively restores high proliferation rates, as observed in bulk nonstem cancer cells (Fig. 1). When mixed and inoculated together, CSC‐like and nonstem cancer cells exhibited faster tumor growth compared to CSCs injected alone, supporting the notion that CSCs drive tumorigenesis through nonstem cancer cells. The influence of Etv1 SUMOylation status on CSC functions endorses the concept of SUMOylation‐dependent transcriptional programs, or SUMO switcher genes, for transcription factors with oncogenic potential (*e.g*., Myc), as initially proposed by Kessler *et al*. [4]

Upon evaluating the effect of ectopic Etv1 constructs (SUMO‐conjugated or resistant) on restoration of colony formation capacity in Ube2i knockout mammary cancer cells, the authors suggested broader implications of the SUMOylation machinery in regulating tumor‐initiating functions, beyond Etv1's contribution. The observation of MMTV‐Neu transgene silencing in Ube2i‐deficient cells prompted the authors to suggest that SUMO‐conjugated Etv1 is a downstream driver of the Neu oncogenic pathway, since constitutive SUMO‐conjugated mutant Etv1 restored tumorigenesis independent of Neu expression. The findings further indicate that SUMOylation plays a role in promoter regulation, either via alteration of transcription factors and cofactor interaction patterns or structural modifications of the chromatin. For instance, histone SUMOylation was suggested to influence chromatin structure and dictate cell state transitions, although this topic remains underexplored. Current evidence indicates that linker histone H1 SUMOylation is essential to trigger and maintain heterochromatin condensation in the transition from totipotency to pluripotency in embryonic stem cells (ESCs) (Fig. 1) [5]. The SUMOylation pathway is highly dynamic during early embryogenesis, and its suppression in mouse embryos increases genome‐wide DNA hypermethylation, supporting the emergence of cell diversity [6]. Moreover, SUMO was shown to maintain heterochromatin integrity via H3K9 methylation in ESCs by regulating Kap/Setdb1 repressive complexes [7]. Together, these observations raise the question of whether SUMOylation in cancer might prevent the adoption of chromatin signatures associated with lineage commitment and differentiation. As CSCs are currently widely regarded as a dynamic cell state rather than a cell type, the role of SUMOylation in tumor cell plasticity invites closer examination.

Recent literature suggests that SUMOylation plays a role in modulating the tumor microenvironment (TME), in part by impeding adaptive antitumor immunity [8, 9]. Although SUMOylation in cancer has been studied primarily on malignant cells where it is highly active, *in vivo* inhibition of the cascade in syngeneic models of colorectal cancer, pancreatic ductal adenocarcinoma (PDAC), and B‐cell lymphoma showed a substantial impact on natural killer and T‐cell dynamics in the TME, increasing intratumoral immune cell infiltration and activation [8]. In a mouse orthotopic PDAC model, the *in vivo* inhibition of SUMOylation by TAK‐981, an SAE inhibitor, significantly decreased tumor growth, but only in the presence of functional T and B lymphocytes. Indeed, engrafting such PDACs in Rag1 knockout recipients abolished the survival benefits from TAK‐981 treatments, supporting a role for immune cells within the TME in the tumor‐suppressing effects of SUMOylation blockade [8]. It was demonstrated that inhibiting the cascade upregulated the expression of T cell activation genes and reduced terminal exhaustion markers, leading to an increased proportion of memory T cells in tumors. Similarly, active SUMOylation was linked to immune evasion in B‐cell non‐Hodgkin lymphoma, where pharmacological inhibition of SUMOylation induced the MHC‐I antigen presentation machinery [10]. Considering that the presence and frequency of CSCs are tightly linked to immune evasion and poor response to immunotherapies [11, 12], further investigations may reveal that SUMOylation activity in CSCs is critical for repelling and blocking the activation of tumor‐targeting immune cells (Fig. 1).

## 2 Harnessing SUMOylation in drug discovery

Knowledge of the SUMOylation cascade has enabled the development of pharmacological modulators of the pathway from different angles, representing promising avenues for cancer therapy or prevention. So far, potent inhibitors of the E1 and E2 enzymatic steps of the SUMOylation cascade have been successfully developed, including the E1 disruptors TAK‐981, McM025044, and ML‐792, and the E2 blockers 2‐D08 and Spectomycin B1. Such next‐generation compounds exhibit superior potency and selectivity compared to gingkolic and anacardic acids, and some have reached clinical testing [13]. By contrast, the SUMOylation balance can be pharmacologically tipped by blocking deSUMOylation using SENP inhibitors (Momordin, GN6958) [13]. While pan‐SUMO inhibition in tumorigenic cells is currently pursued to capitalize on its cancer‐specific dependency, other CSC‐targeting small molecules, such as CWP232228 and YB‐0158, are taking advantage of active SUMOylation to facilitate anti‐CSC mechanisms, including the nuclear translocation of Sam68, which consequently inhibits canonical WNT gene networks in leukemia and colorectal cancer [14, 15]. Understanding the specific mechanisms underlying SUMOylation dependency in different cancer and healthy tissue contexts will be crucial for the successful utilization of pharmacological pathway modulators in therapeutic regimens.

## 3 Concluding remarks

The work by Li *et al*. is an example of a sophisticated and detailed investigation necessary to understand and precisely define SUMO‐dependent mechanisms of tumorigenesis and cancer stem cell maintenance. Importantly, inhibition of SUMOylation might lead to the transition of CSCs into nonstem cancer cells, known to be more susceptible to standard cytoreductive therapies in the clinic and can be leveraged in potential combination approaches. While SUMOylation dependency is widely attributed to cancer, further work is required to contextualize the findings obtained in mammary tumorigenesis and understand their applicability in other malignancies. For instance, Lopez *et al*. demonstrated that Ubc9 haploinsufficiency has a pro‐oncogenic effect in an intestinal tumorigenesis mouse model [16]. Such a potential context‐specific tumor suppressive effect of intact SUMOylation machinery raises awareness for future anticancer therapies based on the untargeted pharmacological inhibition of this cascade. It will be crucial to fully unravel the balance of specific pro‐ and antitumorigenic SUMOylation‐dependent mechanisms in malignancies and healthy tissue homeostasis, especially regarding systemic pharmacological modulation of the cascade. Understanding the contributions of tumor cell‐intrinsic and microenvironment‐dependent processes that lead to tumor growth inhibition, as well as the potential short‐term and long‐term adverse effects of this therapeutic approach, is critical for the successful application of SUMOylation modulators in the clinic.

## Conflict of interest

The authors declare no conflict of interest.

## Author contributions

VY wrote initial draft and created the figure. YDB supervised the writing and conceptual design of the article. VY and YDB cowrote the final version of the article.
